# Less Can Be More: RNA-Adapters May Enhance Coding Capacity of Replicators

**DOI:** 10.1371/journal.pone.0029952

**Published:** 2012-01-23

**Authors:** Folkert K. de Boer, Paulien Hogeweg

**Affiliations:** Theoretical Biology and Bioinformatics, Universiteit Utrecht, Utrecht, The Netherlands; Semmelweis University, Hungary

## Abstract

It is still not clear how prebiotic replicators evolved towards the complexity found in present day organisms. Within the most realistic scenario for prebiotic evolution, known as the RNA world hypothesis, such complexity has arisen from replicators consisting solely of RNA. Within contemporary life, remarkably many RNAs are involved in modifying other RNAs. In hindsight, such RNA-RNA modification might have helped in alleviating the limits of complexity posed by the information threshold for RNA-only replicators. Here we study the possible role of such self-modification in early evolution, by modeling the evolution of protocells as evolving replicators, which have the opportunity to incorporate these mechanisms as a molecular tool. Evolution is studied towards a set of 25 arbitrary ‘functional’ structures, while avoiding all other (misfolded) structures, which are considered to be toxic and increase the death-rate of a protocell. The modeled protocells contain a genotype of different RNA-sequences while their phenotype is the ensemble of secondary structures they can potentially produce from these RNA-sequences. One of the secondary structures explicitly codes for a simple sequence-modification tool. This ‘RNA-adapter’ can block certain positions on other RNA-sequences through antisense base-pairing. The altered sequence can produce an alternative secondary structure, which may or may not be functional. We show that the modifying potential of interacting RNA-sequences enables these protocells to evolve high fitness under high mutation rates. Moreover, our model shows that because of toxicity of misfolded molecules, redundant coding impedes the evolution of self-modification machinery, in effect restraining the evolvability of coding structures. Hence, high mutation rates can actually promote the evolution of complex coding structures by reducing redundant coding. Protocells can successfully use RNA-adapters to modify their genotype-phenotype mapping in order to enhance the coding capacity of their genome and fit more information on smaller sized genomes.

## Introduction

It has been generally accepted that replicators during the early stages of evolution, most likely formed an ‘RNA world’ [1], [2], where life consisted primarily of self-replicating RNA-molecules with catalytic properties, so called ribozymes. RNA is considered to be extremely versatile and flexible due to the genotype-phenotype mapping between genetic information and function based on the folded structure. This results in properties advantageous for evolvability, such as a redundant mapping and neutrality [3]–[5], making RNA an ideal evolvable molecule [6], and moreover: an appropriate model to study evolutionary processes in general [7], [8].

Surprisingly, among the non-coding RNAs found in contemporary organisms, many are actually used to effectively influence the mapping between genetic content and the function it encodes (being other RNAs or proteins). That is, functional and structural inter-relationships of macro-molecules are widespread [9], and a variety of fundamental cellular processes are governed by molecular chaperones [10]–[12]. Also, the genotype-phenotype mapping can be flexible in the way functional information is stored on a genome. The most notorious example in these is alternative splicing (eg. multiple proteins coded on a single gene), which is commonly used and allows for information to be stored much more economically, and a more varied proteome from a genome of limited size [13]. Building on these observations, the current study will investigate the evolution of (early) replicators, when provided with the possibility to acquire a simple abstraction of a molecular tool for such self-modification of their genotype-phenotype mapping.

In contemporary life, these RNA-RNA modification machineries range in complexity from simple physical obstruction of translation by complementary base-pairing, to chemical modification, insertions and deletions of nucleotides by advanced RNA-editing processes, in which RNAs and proteins work together [14] or RNAs are modified under guidance of snoRNAs, which associate with a set of proteins [15]. Antisense RNA is found to bind to mRNA, forming double-stranded RNA that is enzymatically degraded[16], [17], miRNAs and siRNAs are found to cause genes they target to be methylated, thereby decreasing or increasing transcription of those genes [18]–[20] and the number of known self-modification machineries keeps expanding. Perhaps most striking is the regulation of gene expression by riboswitches: structured RNAs, able to change their conformational states by binding of small molecules and to regulate several different processes [21]–[24]. Riboswitches interact directly with their effectors and do not require additional factors. Therefore, it has been suggested that these riboswitches represent one of the oldest regulatory systems [21].

We hypothesize that such machinery could be a way for early replicators to cope with the major problem they have to face, known as the information threshold [25]. That is, within error prone environments the number of errors during replication is expected to be extremely high, preventing replicators to increase their genome above a certain size. Hence, replicators during prebiotic evolution may be stuck with small sized genomes, unable to code for error correcting mechanisms, while they need such a mechanism to become longer. This problem is known as Eigen's paradox [25] and raises the question how a system of ‘simple’ RNA-based replicating protocells can evolve the widespread genotypic, and hence phenotypic, complexity, common for contemporary life.

Major attempts to solve the problem of the information threshold have been made in the context of molecular organization [26]–[28]. That is, the evolution of complexity has been studied mostly on the level of replicator-interactions in the context of a predefined or emergent organization of replicating molecules [25], [27]–[30]. In contrast, we are interested how the versatility of RNA-landscapes can be used to enhance information accumulation and we address the evolution of complexity *within* replicating protocells. For this purpose we constructed a model of replicators (ie. protocells), evolving towards a fixed set of arbitrary RNA-structures functional by definition, concurrently avoiding any other (misfolded) structure. Note that, because the genetic content of reproducing protocells is copied at once, we do not study the coexistence of independent replicating RNAs within compartments, as for example in [31], [32].

With respect to the classical problem of the information threshold, it should however be mentioned that it becomes increasingly evident that the information content required for activity is much lower than indicated by the length of replicators [33], [34]. Recently, smaller and smaller RNAs have been found to be functional. Although rather unselective and having weak catalytic activity, five nucleotide long ribozymes are already reported to be able to transcribe multiple products [35] and the study of small self-cleaving ribozymes has revealed that remarkably small RNA units can already achieve high sequence specificity and catalytic efficiency, where the composition of many of the segments has little effect on catalytic activity [34]. This indicates that mutation rates can be substantially less restricting than initially thought, because neutral and compensatory changes tend to dampen the effects of deleterious mutations even more, allowing a relaxed error threshold [33]. Moreover, recently an RNA polymerase has been engineered, capable of accurately synthesizing a wide spectrum of RNA-sequences of up to 95 nucleotides in length [36], and RNAs capable of biochemical catalysis are likely to be quite common in sequence space [37].

Given the above considerations, the common reputation of the information threshold might have to be reconsidered. The dynamics of evolution itself imposes patterns on the coding structure of replicating units [38] and as such we will regard the information threshold not merely as a problem of information limitation, but more as a structuring constraint for evolution, shaping how coding structures and complexity of replicators evolve [30], [39].

In a model-universe where only a small set of conformations of RNA-sequences is considered to be ‘functional’, while all misfoldings are considered to be ‘toxic’, we ask: *can replicators employ self-modification of genetic information to alter their genotype-phenotype mapping in order to evade the information threshold*, and, *how does the information threshold shape the structure of coding of evolved replicators?*

## Results

The evolution of complexity is studied in protocells evolving towards a set of 25 pre-defined ‘functional’ secondary structures. Two distinct sets of 25 target-structures are used (see Methods section). Protocells are a limited set of RNA-sequences (default maximum of 50), which are folded according to the algorithm of the Vienna RNA package [40]. The resulting structure of a sequence is regarded as functional when the coarse grained structure (shapiro structure [41]) matches a target-structure. Fitness given by a structure depends on the distance to the full secondary structure of the target (however, neglecting any dangling ends). The fitness of a protocell is the sum of the fitness given by all its unique functional structures (no dosage effect). All molecules which are not functional (not matching any structure in the target-set), are regarded as toxic and shorten the average lifespan of cells. Note, that in our case the majority (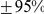 for both target sets) of structures is considered to be toxic, making the role of negative selection against unwanted molecules as important as the selection for reproductive fitness.

**Figure 1:**
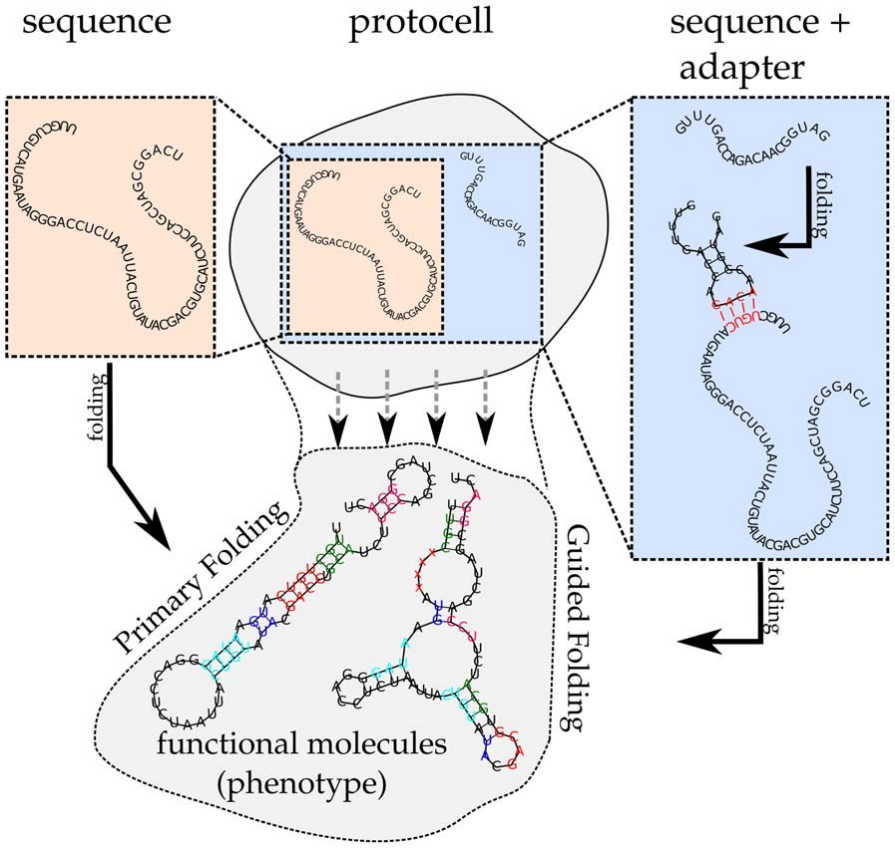
Usage of RNA-adapters. The conformation of RNA-molecules can be guided by the anti-sense binding of RNA-adapters (hairpin). By having an adapter, two distinct structures can be transcribed from the same sequence. Both foldings are regarded as functional and provide fitness for a protocell if matching a target-structure. Adapter change in base-pair configuration is shown by giving corresponding colors to the nucleotides in the formed base-pairs of the primary folding. In this case, the blocking of four nucleotides by an RNA-adapter leads to a different pairing of all nucleotides.

A minimal implementation for adapters is chosen, which could involve either chemical modification or binding induced alteration. Hence, RNA-sequences folding into a secondary structure consisting of a single hairpin-loop, are regarded as ‘RNA-adapters’. Note that early experiments (data not shown) showed that the exact predefined structure is not important for the results. RNA-adapters can bind reversibly, and only if binding free energy is less than 4 kcal/mol. When an adapter binds, it blocks certain nucleotides, which can possibly lead to a change in the conformational state of the bound RNA-sequence. How the new folding is achieved and the impact an adapter can cause on the structural conformation of the bound sequence, is illustrated in Figure 1. Nucleotides of an evolved RNA-sequence are colored in the secondary structure of the adapted molecule, according to the base-pairings formed in the secondary structure without adapter. As shown, under guidance of a co-evolved adapter (blocking only four nucleotides), all nucleotides are base-paired differently.

**Figure 2:**
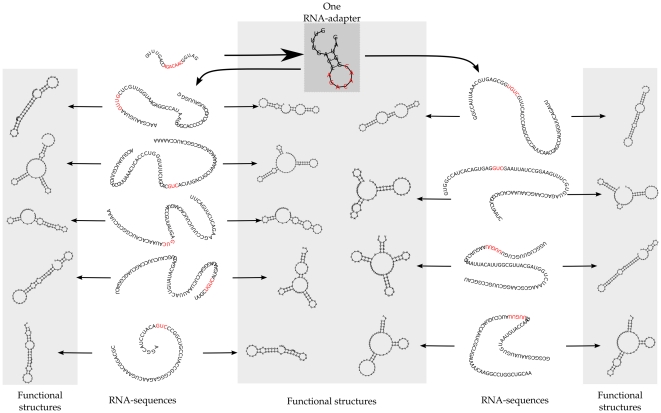
One adapter guides many sequences. Multiple RNA-sequences can be guided differently by one RNA-adapter. Shown are the unfolded RNA-sequences, their original secondary structure (outer panels) and the guided secondary structures (middle panel) when the adapter (shown at the top) binds to the binding sites shown in red. RNA-sequences are co-evolved with the adapter, such that all sequences can code for two distinct functional structures, coding for 18 target structures in total.

The total number of structures a protocell can possibly produce (codes for), defines the ‘phenotype’ of this protocell. With this multi-molecule genotype-phenotype mapping, the incorporation of a single RNA-adapter can thus provide a general self-modifying tool, potentially doubling the functional structures coded for by all other RNA-sequences in the cell. Note however, that such a one-to-many genotype phenotype mapping might potentially lead to a considerable amount of toxicity. Nonetheless, by having RNA-adapters, a protocell can code for more foldings than it has RNA-sequences. This is detailed in Figure 2, showing a protocell (evolved with target set 2, 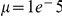, constrained to 10 sequences), which evolved one adapter among multiple RNA-sequences. Depicted are all the structures a single protocell can potentially produce (in the gray panels) from the information it has coded on its genotype. The RNA-adapter (top) is able to bind to ‘regular’ RNA-sequences with the nucleotides colored red. All other RNA-sequences in the protocell have evolved a specific site (in red) where the RNA-adapter can bind.

Population dynamics are given by competition and replication of protocells. Protocells transfer all genetic material (that is, all RNA-sequences) to their offspring at once, under consideration of mutations. In short, we defined a single level evolutionary system and only competition between protocells is considered, not between molecules within a cell. Focus is on the transfer of genetic material (coding for functionality) by replicators. For further details, see the Methods section.

Now we will study the relationship between mutation rates, genome size and usage of coding, given the possibility of RNA-adapters. After identifying the general trends and characteristics of the system we will detail the evolved molecular interaction structures in the protocells and elaborate on the exact nature and implications of adapter usage by protocells. Finally we will scrutinize the evolved coding structures and communication between sequences and RNA-adapters.

### Changing Coding Structures Under Increased Mutation Rates

**Figure 3:**
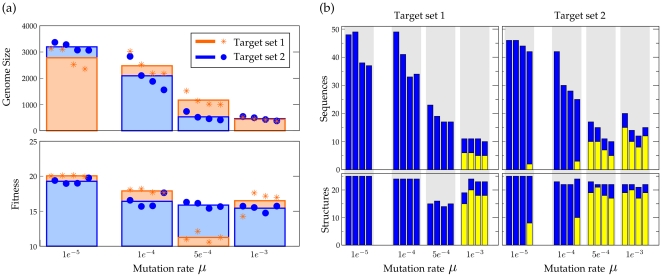
Relation between mutation rates, genome size and fitness. (a) Mutation rates, genome size and fitness of the last common ancestors for the different mutation rates and the two target sets. For each mutation rate four repetitions of a simulation (circles and stars) with a different seed and the average, are shown. Except for target set 1 under 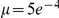, the decrease in genome size does not lead to a comparable decrease in fitness. (b) Number of sequences (above) and the functional structures encoded (below), for both target sets under the different mutation rates. Blue bars are the total number of sequences and structures. Yellow bars depict the number of sequences coding for adapters (above) and functional structures guided by adapters (below). While under low to moderate mutation rates almost all functional structures are acquired without the need for RNA-adapters, under high mutation rates, they are commonly incorporated into the coding structure of protocells. This transition explains the relation between mutation rates, genome size and fitness, observed in (a).

Genome size (number of nucleotides or the sum of the length of all RNA-sequences in a protocell) and fitness of evolved protocells is studied in simulations under increasing mutation rates. For the two target sets, four simulations are performed with the mutation rates 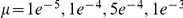. In each simulation, the last common ancestor is taken, of which length and fitness is given in Figure 3a. Genome-size decreases under increased mutation rates for both target sets, indicating the size-limitations posed by the information threshold. However, under most severe mutation rates, fitness is restored to values comparable to the fitness of protocells under low mutation rates. For target set 2, this already happens with mutation rate 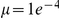: fitness is kept more or less constant, while the decrease in genome size is considerable. For target set 1, fitness decreases under mutation rate = 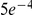 and only under the highest mutation rate, fitness is restored. Thus, overall, under increased mutation rates the protocells are able to achieve fitness with much shorter genomes. This suggests a change in the way information is coded on the genome, compensating smaller genomes by employing a different coding structure. If mutation rates are even more increased (to 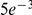 and higher, data not shown), fitness drops to values fluctuating approximately between 1. and 5. and the functional structures cannot be kept over time. Almost random drift of functions and fitness indicates that the protocells are over the information threshold.

The change in coding structures under high mutation rates is confirmed, on a general level, in Figure 3b. Depicted are the number of RNA-sequences a genome encodes and the number of structures a protocell can produce with this. As there is no dosage effect assumed, multiple copies of the same function do not give any fitness advantage. However, under moderate mutation rates, redundant coding is used as a backup for fitness-providing RNA-sequences and protocells have more RNA-sequences than functional structures.

The protocells in Figure 3b which use RNA-sequences to code for adapters, correspond to the small sized genomes of protocells in Figure 3a. In these protocells, the majority of functional structures are produced under guidance of RNA-adapters and a set of functional structures of comparable size is achieved with only a fraction of coding size, used under moderate mutation rates. Also note the only simulation with target set 2 under 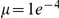, where RNA-adapters are incorporated, enabling this protocell to code for more functional structures than the other evolved protocells under this mutation rate, and a higher fitness.

These observations show that mutation rates impose a restriction on the used coding length during evolution, similar to that described in other work [39]. This restriction in usable genome length forces protocells to gain functionality despite the limitations in genome size they can maintain. In other words, coding capacity can be enhanced by the use of adapters. However, in the studied simulations, adapters are used only under increased mutation rates.

### Adapter Usage

**Figure 4:**
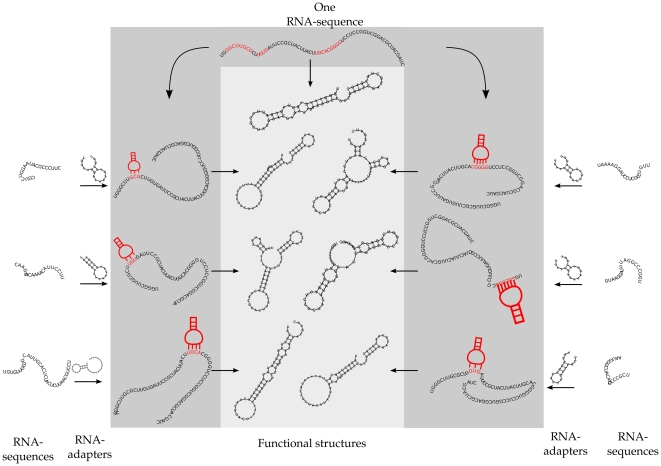
Many adapters guide one sequence. Having multiple adapters in a protocell, can lead to an extreme concentration of different functions coded for by an RNA-sequence. The RNA-sequence shown at the top codes for seven distinct functions in target set 2. To the left and right of the middle panel, the six RNA-sequence which code for an adapter and their secondary structures (always a single hairpin loop) are shown. These adapters bind each to a different site, guiding the RNA-sequence into a different folding.

The molecular composition in protocells under severe mutation rates in Figure 3, show more than twice as many functional structures, as RNA-sequences used to code for these structures. The mechanism by which this is achieved, is in the line of Figure 2 and shown in Figure 4. Only a subset of the RNAs on a genome evolved under 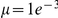 (with target set 2) are displayed. With six different adapters, one ‘regular’ RNA-sequence maintains enough information to code for seven distinct functional structures. Next to the primary function obtained by folding the original sequence, six binding sites can be identified, specific for the six RNA-adapters (colored red, although with some overlap). By binding to these binding sites, different RNA-adapters act as specific modification machinery, each changing the minimal energy conformation of the sequence in their own specific way. The minimum free energy folding of the modified sequences is found to be comparable to the original folding (without the free energy of the adapter-binding itself taken into account).

Concluding, protocells under high mutational pressure can compensate for a small genome size by adopting complex molecular coding structures, increasing the coding capacity of RNA-sequences by evolving general and/or specific code-modification machinery. The code used between such RNA-adapters and regular RNA-sequences play a key role in this and will be studied next.

### Arbitrary Coding used for Molecular Communication

**Figure 5:**
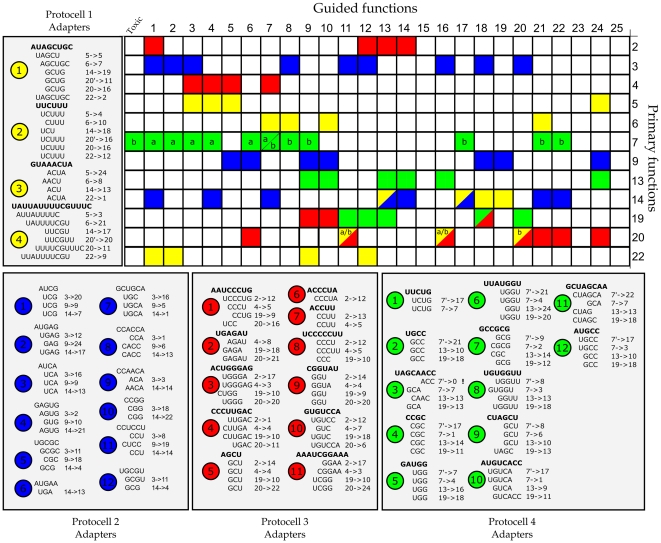
Evolved codes of adapter-sequence interaction sites. Matrix depicting the transformation of primary functions into other functional conformations by guidance of adapters. Colors correspond with the best protocells evolved in four duplicate simulations (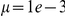, target set 2), only differing in random seeds. All simulations evolve their own specific molecular structure to code for information on their genome. Moreover, for all transformations, the exact code used (bounded stretch of nucleotides) is given. Rows with letters represent multiple RNA-sequences (within the same protocell) which share the same primary function, however they differ in the set of guided functions. The numbers of functions correspond to the secondary structures depicted in the method section (Figure 7), counted from the upper left.

In our model, both sequence and adapter have to evolve a sequential ‘code’ to be able to correctly base-pair adapters and RNA-sequences in a protocell. The arbitrariness of such evolved codes is exemplified in Figure 5. All RNA-sequences, the functions they encode, and the binding site of adapters are displayed for four simulations, only differing in random seeds, for target set 2, 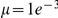 (see Figure 3). Each row of the matrix represents an RNA-sequence with its realized primary function and each colored grid point corresponds to the function this RNA-sequence codes for under guidance of an adapter. Note that grid points with letters represent RNA-sequences in the same protocell with the same primary function, suggesting the independent specialization of self-modification of two copies formed by duplication.

It is evident that, although evolved under exactly the same global circumstances, each simulation produces protocells with a distinct combination of primary and guided functions on their RNA-sequences. Not only the combinations of functions over sequences is evolved at liberty, but also the coding used for molecular communication is seemingly assigned at random. Notable is, that out of the 136 here reported function transitions only one leads to a toxic structure (*green* adapter 3). This shows that not only functional interactions did evolve, but also that all other interactions which produce toxic structures are successfully avoided.

### The Role of Redundancy and Complex Coding Structures

Protocells in the described simulations can have up to 50 RNA-sequences, while both target sets contain only 25 possible functional structures. When not restricted by the information threshold, protocells are essentially free to “choose” how they code for their phenotype. Therefore, it could be hypothesized that under low mutation-rates protocells do not need coding capacity enhancement, because they can maintain genome sizes large enough to code for all functions. However, analysis of the redundancy of coding indicates that this is not the whole story.

**Figure 6:**
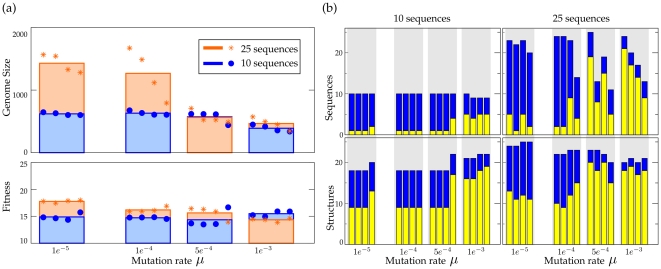
Cell-size limitation. (a) Genome size and fitness of the last common ancestors for simulations with the number of sequences limited to 10 and 25 sequences. For each mutation rate four repetitions of a simulation (circles and stars) with a different seed and the average, are shown. Note that results under high mutation rates differ the least, suggesting a ‘natural’ upper bound for genome size. (b) Number of sequences (above) and the functional structures encoded (below), for both limitations with the four mutation rates. Blue bars are the total number of sequences and structures. Yellow bars depict the number of sequences coding for adapters (above) and functional structures guided by adapters (below). By restricting the number of sequences, redundancy is suppressed and adapter usage is promoted.

Figure 6 shows the results for simulations where the number of RNA-sequences in a protocell is restricted independent of mutation rates. Evolving towards target set 2, protocells are limited to having a genome at most consisting of 10 or 25 RNA-sequences. In the first case, this boils down to the necessity of using RNA-adapters in order to gain maximum number of functional structures and fitness. In the latter, cells can code for full functionality without adapters, but are restricted in the redundancy of RNA-sequences.

As shown in Figure 6, such a limitation results for almost all simulations in the use of adapters. Thus, we can conclude that limiting redundancy facilitates the evolution of self-modification machinery under low mutation rates. However, the exact implementation of RNA-adapters is still dependent on mutation rates. While under moderate mutation rates, a majority of functional structures is still coded for as the primary function on a sequence, under severe mutation rates this ratio is inverted and most functional structures are coded for under guidance. That is, the degree of enhancement of coding capacity still depends on mutation rates. With increasing mutation rates, phenotypes of protocells are coded for on smaller sized genomes. Moreover, the convergence of genome sizes under high mutation rates, suggest that the upper bound for genomes is determined by mutation rates instead of external limitation.

Interestingly, in the case of the maximum of 10 RNA-sequences, only under higher mutation rates more than one adapter is used, which results in the unexpected reversal: protocells under severe mutation rates obtain the highest fitness. This is, most likely, because the adaptive valley, formed by a decreased fitness (eg. a functional structure has to be sacrificed to code for an RNA-adapter), and increased toxicity (by the introduction of a new RNA-adapter), cannot be crossed.

Thus, redundant coding impedes the evolution of self-modification machinery on a genome, in effect restraining the evolvability of coding structures. However, when redundancy is restricted (by either mutation rates or external limitation), coding capacity can be enhanced by adopting a complex coding structure, guided by adapters. Hence, an increase of mutation rates not only force protocells to employ complex coding structures, but also facilitates the evolution of adapters.

But what happens when the protocells manage to decrease mutation rates? We observe that when subjecting a population initially evolved under high mutation rates, to low mutation rates, the adapter machinery is kept. However genome sizes can increase due to the reduced mutational pressure. This results in a coding structure which is not observed to evolve in simulations with constant low mutation rates: whereas redundancy prevents adapters to evolve, redundancy can evolve when adapters already have evolved. Hence, these results show that the limits posed by the information threshold are in fact responsible for the development of a compact and ‘complex’ structure of coding, observed in our model-universe.

## Discussion

Not only in present day organisms RNA-modification has an important role. It has been claimed that at least a simple form of alternative splicing may already have been present in the unicellular ancestor of plants, fungi and animals [42]. RNA has also been suggested to have regulated and controlled cellular life all the way from the origin of life [17]. Primitive chaperones could well have been present in the ‘primordial soup’ [43] and one of the oldest regulatory systems is represented by structural RNA regulators in the form of riboswitches [21]. We studied the evolution of coding structures in RNA-based protocells when given the possibility to code for adapters, representing a simple abstraction of such RNA-RNA modification machinery.

Results show that the use of adapters in combination with the properties of RNA, allow for the evolution of a one-to-many genotype-phenotype mapping, where genetic information is combined and multiple functional structures can be generated from a single sequence. Self-modification of the genotype-phenotype mapping of a replicator can thus effectively pave the road for more complex coding structures to evolve. Hence, the proposed mechanism could be a way to increase the functional repertoire and complexity of early life replicators with only small sized genomes, evading the limits posed by the information threshold, while maintaining complex coding structures. By successfully enhancing code capacity, the mutational landscape of a replicator is altered and more advanced mechanisms, as found in contemporary organisms (like RNAs chaperoning proteins or differently translated RNA-molecules from DNA by using pre-mRNA and alternative splicing), may come within reach.

However, we should mention that in the light of prebiotic evolution, ‘realistic’ protocells would not be able to transmit all the information of a parental protocell to its daughter protocells at once, as is assumed in our model. That is, here we study the information content of single level replicating systems, as “cells” which contain several RNA-sequences. This is a single level process, because the replication of the RNAs is directly coupled to the reproduction of the containing cells (According to [44] the correct phrase in this case would actually be ‘reproducer’). This can be seen as a shortcoming of our approach, as in early evolution it should be expected that RNAs replicate independently and that these replicating RNAs are (randomly) divided when protocells split. Such unequal distribution of templates in daughter cells leads to a two level selection process, where selection on the cell level may counteract selection on the replicator level, as first shown in the stochastic corrector model of Szathmáry and Demeter [27], and further studied by e.g. [31], [32]. However, such dual level selection does not solve the Eigen paradox [31], [45]. Neither does linkage of templates enabling equal distribution of the templates in daughter cells, as already noted by Eigen [46]. We choose to study a simpler model with linked RNA-sequences, to be able to focus on the potential increase of functional information carried by protocells due to “smart” coding. Challenge remains to investigate if the observed results can also be achieved in multi-level replicating systems or when RNAs are assumed to be the main replicators.

What we can learn from this model, is that the limits to the versatility and possibilities of the evolution within an RNA-landscape have not yet been reached. Rather than solving the information threshold, our results show that by including evolvable molecular interactions, the relation between genomes and phenotype can go beyond the (physically) determined genotype-phenotype mapping. The way information is accumulated and how coding structures are shaped, depends on limitations posed by the information threshold [39]. That is, enhanced code capacity given by adapter based genotype-phenotype mapping modification, is observed only under high mutation rates. The explanation for this is two-fold. First, the information threshold forces replicators to adopt a compact and ‘smart’ structure of coding to alleviate mutational pressure. Secondly, the information threshold reduces the length of coding that can be maintained, which leads to less redundancy on a genome. This allows adapter-molecules to be more easily incorporated in the genotype because the likelihood of toxicity is reduced. The role of toxic molecules is most obvious by regarding the linkage between mutation rates and genome-size. While single mutations can affect fitness only through changes in secondary structure, the main (negative) effect is given by the toxicity of misfolded structures. Such a relation between mutations and toxicity is described for protein evolution, where it has been shown that protein structures may be remarkably tolerant to many mutations [47], while unstable essential proteins result in lethality through misfoldings [48]. Our model shows that when the toxicity of such misfolded molecules is taken into account, redundant coding impedes the evolution of self-modification machinery on a genome, in effect restraining the evolvability of coding structures. However, under increased mutation rates or an external limitation of the number of RNA-sequences in a protocell, redundant coding is suppressed and coding capacity can successfully be enhanced by the use of self-modification machinery. This increased coding capacity could be exploited when mutation rates are lowered to code for novel machinery, eg. repair mechanisms and therewith lowering mutation rates even further.

Finally, we showed that an arbitrary coding used for “communication” between adapters and other sequences is readily evolved. Such arbitrariness of biological coding is recently nicely demonstrated [49]. The randomness of the coding used for communication between adapters and sequences is also in accordance with the recent finding of low sequential conservation of sRNA-mRNA interaction sites [50].

However, despite the power of the described self-modification machinery and its arbitrary code, there is the potential danger of interference between the functional structure of molecules and the coding used for adapters. Interference between different functions encoded on one RNA does occur in present day organisms. For example, the structure of RNA will constrain the variability and evolution at the protein level (as shown for the evolution of HIV [51]) and the other way around, the conservation of a protein code in an RNA sequence will put restrictions on its potential to evolve certain secondary structures [52]. Moreover, folding of proteins is an especially error-prone process that requires help from molecular chaperones, and it has been suggested that chaperoning was not only a common feature of several ancient proteins and RNAs, but that it was also necessary for the evolution of modern protein enzymes [43].

### Conclusion

This study sheds new light on the relationship between mutation rates and replicator complexity. Allowing for the self-modification of molecular functions by the binding of anti-sense molecules gives replicators a new degree of freedom in how they ‘choose’ to code for their phenotype. Results show that protocells successfully employ RNA-adapters, to gain functionality, and/or to decrease genome size under high mutation rates. The avoidance of unwanted interactions (those which lead to toxic misfoldings), make redundancy a barrier for the evolution of code capacity enhancement. In effect, by the limitation of such functional redundancy, mutation rates pave the road for incorporating self-modification machinery on a genome. We show that, when taking RNA-RNA modification interactions into account, the information for multiple functional structures can be coded for on a sequence, and by enhancing coding capacity, the limitations posed by the information threshold can be effectively alleviated. Hence, already when taking (simple) molecular sequence-modification tools into account, the information threshold has to be considered more as a structuring constraint, shaping the structure in which phenotypes of replicators are coded for on a genome.

## Methods

We model the evolution of a population of protocells. Each protocell consists of a genome, which is a set of RNA-sequences, coding for RNA-structures (molecules).

The multi-molecule genotype-phenotype mapping consists of all RNA-sequences being folded (based on the the Vienna RNA package [40]), providing a phenotype of all the structures a cell can possibly produce from its genotypic information. Molecules consisting of a single hairpin-loop (Shapiro notation: (((H)S)R)) are defined to be “RNA-adapters”. RNA-adapters can guide the folding of other RNA-sequences by base-pairing. If a protocell has at least one RNA-adapter, it is calculated for all other sequences in the protocell if the adapter(s) can bind. The hairpin of an RNA-adapter can only bind to other sequences in reversed orientation, that is from the 3′-end to the 5′-end of the loop. However, a bounded stretch of nucleotides should always be followed with a non-bound nucleotide on the 5′-end. The lowest free energy is calculated which can be obtained by (reversed) binding of the adapter antisense without gaps to the structure under guidance. Free energies are calculated according to the free energy contributions from stacked base-pairs as in the Vienna package [40]. Only free energy smaller than −4.0 kcal/mol are considered suitable and the actual bound stretch of nucleotides is blocked (substituted by an ‘x’), after which the RNA-adapter releases again. The evolved adapter-interactions have much lower free energy bindings (being only small stretches). If multiple binding sites with exactly the same free energies are determined, the adapter binds only to most upstream site of the RNA-sequence.

When an adapter can bind, the structure under guidance is folded, based on the sequence with certain nucleotides blocked. An example of a protocell with two structures of which one is an RNA-adapter is given in Figure 1 (results section). Note that if a protocell contains multiple RNA-adapters, this can possibly give rise to several more functions of the original RNA-sequence (for example, see Figure 4). RNA-adapters cannot code for other functions or be bound by other adapters. All functions (that is, the sum of all primary and guided structures) produced by all the RNA-sequences, are considered. In the case of the example in Figure 1 the protocell would thus have two functions (one primary and one guided function), Figure 4 depicts 7 functions.

Fitness is based on the number of predefined target structures a protocell can potentially transcribe. That is, if a folding of a sequence matches the course grained structure in Shapiro notation [41] of a target-structure, the distance of the secondary structures without dangling ends (based on their alignment), relative to the length of the target-structure is computed. Dangling ends are discarded from fitness to provide some flexibility in coding for multiple target-functions of different length. The actual fitness which is gained by a protocell by having a folding then is 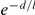, with 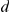 being the distance to, and 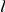 the length of the secondary target-structure with the same shapiro structure. Note that in this way, “functionality” of molecules is defined coarse grained and the specificity of a structure (matching the full secondary structure) tells how well a function is performed. Each function can be performed only once, so within a protocell only the structures matching a target-structure best are considered for fitness. Foldings of sequences which do not match (coarse grained) any target-structure, are considered toxic. Here, toxicity is implemented as an increased death rate. However, the implementation of toxicity does not matter for the reported results. Experiments where misfoldings lead to a decreased overall fitness for replication instead of an increased death rate did show the same trends (see Figure S2).

**Figure 7:**
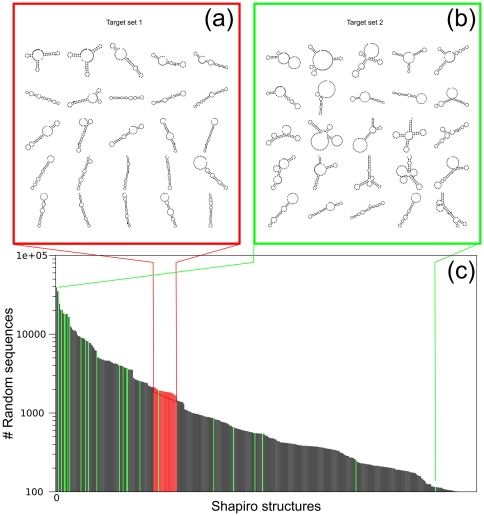
Secondary structures of the used target sets. The different secondary structures which are defined to be functional in our model universe. (a) Target set 1, (b) target set 2, (c) the number of secondary structure (from a million random RNA-sequences of length 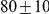) which fold into a specific shapiro structure. In red are the shapiro structures of target set 1, in green those from target set 2. Note that only a part of the distribution is shown. A total of 2130 Shapiro structures with an abundance of less then 100 secondary structure is not shown.

The secondary structures of all targets are depicted in Figure 7. To obtain target set 1, a million random RNA-sequences of length 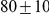 were folded and the abundance of their coarse grained structure was counted. With the most common structures as target set, the chance for replicators to have a considerable amount of functional molecules at initialization is very high. Therefore, to be able to study an evolutionary trajectory towards a set of targets, we choose to pick a set of structures which are less abundant. Together, the frequency of random sequences having a coarse grained structure which is in this target class is 4.65%.

Target set 2 consists of ‘real-life’ RNA-structures predicted to be functional, picked from the fRNA-database (http://www.ncrna.org/frnadb/). These structures were (hand)picked with ‘different’ as the most important criterion. Note that the major objective of this set is to show the independence of our results towards choice of target. The frequency of random sequences (length = 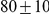) folding into this target set is 5.9%. The distribution of abundance of both target sets is shown in Figure 7 c. It can be seen that the total abundance is comparable, however the structures of target set 2 are less localized in the distribution, compared to target set 1. Note that also the least frequent structures of target set 2 are evolved in several simulations.

Replication is asexual and achieved by copying an entire genome into a new protocell. However, during copying, complete RNA-sequences can be duplicated or deleted with probability 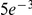 per RNA-sequence. On the level of the molecules, a part of the sequence (random length) can be either duplicated or deleted, both with a probability of 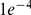. Finally, single nucleotides can be deleted, inserted or mutated, with probability 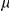. When discussing the role of mutation rates, this considers the 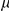 on the level of the nucleotides only.

Our model universe comprises a spatially explicit population of protocells. Each of the protocells can inhabit a position on a 50×50 grid. The boundaries of the grid are toroidal and protocells can interact only within their local neighborhood, consisting of their own position and the eight adjacent positions (Moore neighborhood). Empty squares of the grid act as resource. Competition for an empty square is between all neighboring protocells. Between competing protocells, the probability that one is selected, is proportional to its 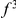 where 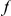 is the sum of all fitness provided by the target structures it can produce.

We used this spatial setting as default. However, when the population is mixed at each time step, similar results are observed: the evolution of adapters can increase coding capacity at high mutation rates, however adapters evolve less frequently (see Figure S1). Thus, although a spatial setting has a positive effect over the well-mixed case, as we tend to see in general, it is not necessary for the main results reported in this paper.

Finally, all protocells decay with the probability of 0.4 plus an extra 0.02 for each non-functional folding it contains. Simulations are started with a population inhabiting 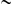
40% of the grid with protocells containing five random RNA-sequences of length 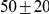 (note that this is below the average length of the target sets). All reported simulations are run for 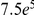 time steps.

### Observables and Ancestor Lineages

Every 1000 time steps statistics on the population are recorded. The evolutionary trajectory of a simulation is best studied and visualized by determining the ancestor lineage. That is, all protocells during evolution carry a unique identifier and the identifier of its parent. After a simulation has finished, the protocells inhabiting the last population can be traced back and all ancestors (out of the recorded populations) leading to this last population can be identified. This gives us the ancestor lineage, which in hindsight contains all protocells which lead to the dominant genetic content of future generations.

## Supporting Information

Figure S1 **Relation between mutation rates, genome size and fitness in a well-mixed population.** We used spatial embedded populations as a default. However, in the current study spatial embedding is not necessary. When the population is mixed at each time step, similar results are observed: although adapters evolve less frequently, the incorporation of adapters still allows protocells to modify their genotype-phenotype mapping, enabling them to increase coding capacity at high mutation rates. Shown are mutation rates, genome size and fitness for the last common ancestors of four simulations for the different mutation rates with target set 2 in a well mixed population. Number of sequences is limited to 25. For each mutation rate four repetitions of a simulation (stars) with a different seed and the average, are shown. Upper squares point out those simulations which use adapters to modify their genotype-phenotype mapping. The evolution and incorporation of adapter-usage is observed less frequently (compare with results of Figure 6 of the main text). Therefore on average, genome size decreases less and fitness decreases more compared to the spatial setting. However when adapters are evolved, this leads to considerable higher fitness on smaller sized genomes, comparable to the spatial case.
(TIFF)

Figure S2 **Reproductive toxicity.** To exclude dependency of our results on the implementation of toxicity as an increase in death rate, experiments are conducted where toxicity is implemented as a negative effect on reproductive fitness. Every misfolding leads to a decrease of 0.2 in fitness. Shown are mutation rates, genome size and fitness for the last common ancestors of four simulations for the different mutation rates with target set 2, number of sequences is limited to 25. For each mutation rate four repetitions of a simulation (stars) with a different seed and the average, are shown. The squares on top depict if adapters are used in that specific simulation. The same trends are observed as in the default (compare with results of Figure 6 of the main text). This shows that the results do not depend on our implementation of toxic misfoldings.
(TIFF)
