# Supplementary figures and images for: Less Can Be More: RNA-Adapters May Enhance Coding Capacity of Replicators
Source: PLoS One. 2012 Jan 23;7(1):e29952. doi: 10.1371/journal.pone.0029952 (PMC3264560; doi:10.1371/journal.pone.0029952)

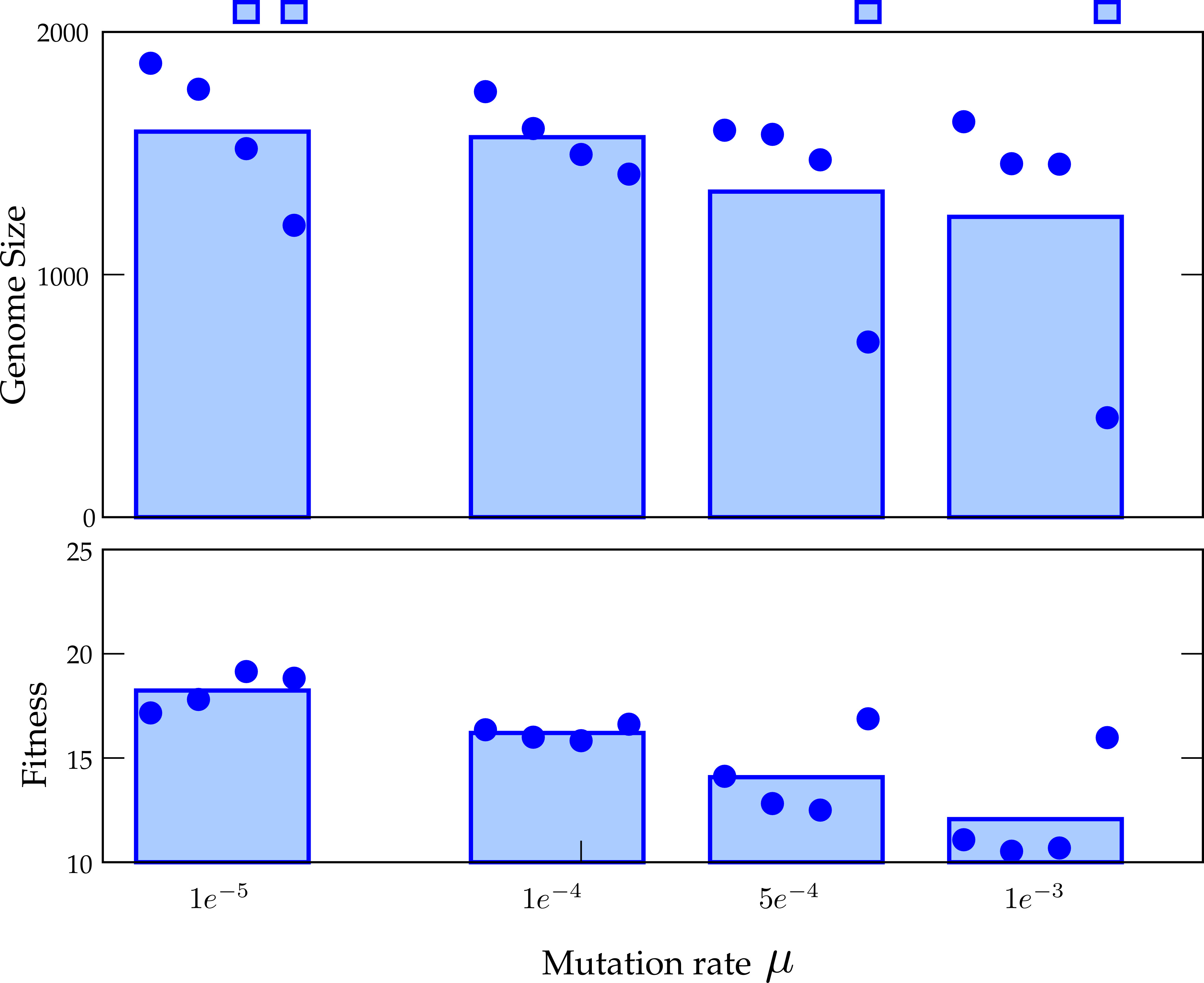

Supplement: Figure S1 — Relation between mutation rates, genome size and fitness in a well-mixed population. We used spatial embedded populations as a default. However, in the current study spatial embedding is not necessary. When the population is mixed at each time step, similar results are observed: although adapters evolve less frequently, the incorporation of adapters still allows protocells to modify their genotype-phenotype mapping, enabling them to increase coding capacity at high mutation rates. Shown are mutation rates, genome size and fitness for the last common ancestors of four simulations for the different mutation rates with target set 2 in a well mixed population. Number of sequences is limited to 25. For each mutation rate four repetitions of a simulation (stars) with a different seed and the average, are shown. Upper squares point out those simulations which use adapters to modify their genotype-phenotype mapping. The evolution and incorporation of adapter-usage is observed less frequently (compare with results of Figure 6 of the main text). Therefore on average, genome size decreases less and fitness decreases more compared to the spatial setting. However when adapters are evolved, this leads to considerable higher fitness on smaller sized genomes, comparable to the spatial case. (TIFF) [file pone.0029952.s001.tiff]

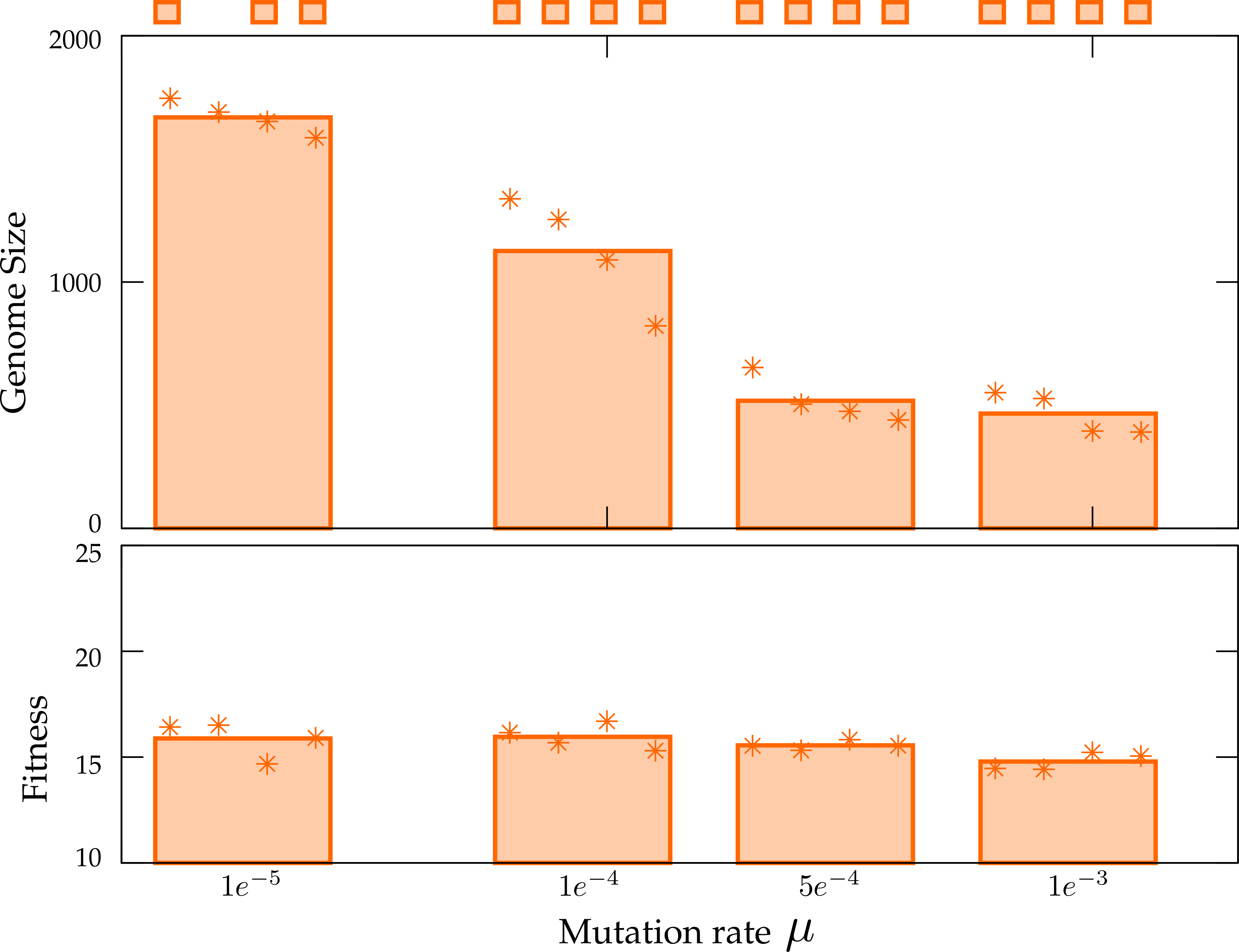

Supplement: Figure S2 — Reproductive toxicity. To exclude dependency of our results on the implementation of toxicity as an increase in death rate, experiments are conducted where toxicity is implemented as a negative effect on reproductive fitness. Every misfolding leads to a decrease of 0.2 in fitness. Shown are mutation rates, genome size and fitness for the last common ancestors of four simulations for the different mutation rates with target set 2, number of sequences is limited to 25. For each mutation rate four repetitions of a simulation (stars) with a different seed and the average, are shown. The squares on top depict if adapters are used in that specific simulation. The same trends are observed as in the default (compare with results of Figure 6 of the main text). This shows that the results do not depend on our implementation of toxic misfoldings. (TIFF) [file pone.0029952.s002.tiff]
